# Laser-assisted surface alloying of titanium with silver to enhance antibacterial and bone-cell mineralization properties of orthopedic implants†

**DOI:** 10.1039/d3tb02481d

**Published:** 2024-04-22

**Authors:** Sotoudeh Sedaghat, Akshay Krishnakumar, Vidhya Selvamani, James P. Barnard, Sina Nejati, Haiyan Wang, David A. Detwiler, Mohamed N. Seleem, Rahim Rahimi

**Affiliations:** a School of Materials Engineering, Purdue University West Lafayette IN 47907 USA rrahimi@purdue.edu; b Birck Nanotechnology Center, Purdue University West Lafayette IN 47907 USA; c School of Electrical and Computer Engineering, Purdue University West Lafayette IN 47907 USA; d Nanovis, West Lafayette West Lafayette IN 47907 USA; e Department of Biomedical Sciences and Pathobiology, Virginia Polytechnic Institute and State University Blacksburg VA 24061 USA

## Abstract

Orthopedic device-related infection (ODRI) poses a significant threat to patients with titanium-based implants. The challenge lies in developing antibacterial surfaces that preserve the bulk mechanical properties of titanium implants while exhibiting characteristics similar to bone tissue. In response, we present a two-step approach: silver nanoparticle (AgNP) coating followed by selective laser-assisted surface alloying on commonly used titanium alumina vanadium (TiAl6V4) implant surfaces. This process imparts antibacterial properties without compromising the bulk mechanical characteristics of the titanium alloy. Systematic optimization of laser beam power (8–40 W) resulted in an optimized surface (32 W) with uniform TiAg alloy formation. This surface displayed a distinctive hierarchical mesoporous textured surface, featuring cauliflower-like nanostructures measuring between 5–10 nm uniformly covering spatial line periods of 25 μm while demonstrating homogenous elemental distribution of silver throughout the laser processed surface. The optimized laser processed surface exhibited prolonged superhydrophilicity (40 days) and antibacterial efficacy (12 days) against *Staphylococcus aureus* and *Escherichia coli*. Additionally, there was a significant twofold increase in bone mineralization compared to the pristine Ti6Al4V surface (*p* < 0.05). Rockwell hardness tests confirmed minimal (<1%) change in bulk mechanical properties compared to the pristine surface. This innovative laser-assisted approach, with its precisely tailored surface morphology, holds promise for providing enduring antibacterial and osteointegration properties, rendering it an optimal choice for modifying load-bearing implant devices without altering material bulk characteristics.

## Abstract 2

Laser-assisted surface alloying (LSA) process to modify orthopedic implant surfaces with Ti–Ag alloy for enhanced antibacterial and osteoinduction properties.

## Graphical abstract

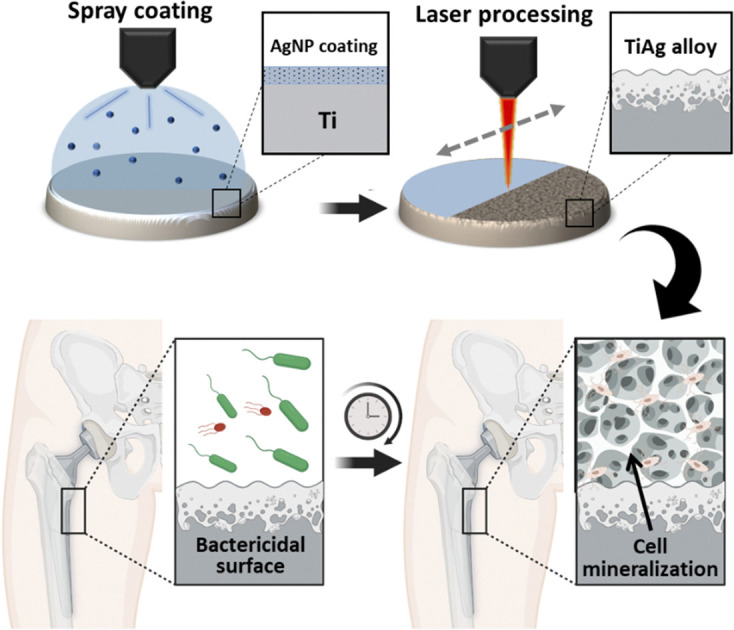

## 1. Introduction

The increasing demand for joint replacement surgeries, driven by an aging population and the rising prevalence of osteoarthritis, has propelled orthopedic implants to the forefront of modern medicine.^1,2^ These implants play a pivotal role in restoring mobility and improving quality of life for individuals suffering from bone and joint complications. Typically, these implants are fabricated from various biomaterials, including stainless steel, cobalt–chromium alloys, and ceramics.^3^ Each material offers distinct mechanical properties, with Young's modulus, a measure of stiffness, playing a critical role in implant success. Among these materials, titanium and its alloys, particularly titanium alumina vanadium (TiAl6V4) alloys, have emerged as a preferred choice due to their exceptional combination of features. With a Young's modulus of approximately 110 GPa, Ti6Al4V offers a balance of strength and stiffness closer to that of cortical bone (∼17 GPa) compared to other materials.^4,5^ This closer match minimizes the risk of stress shielding, a complication where the implant bears the majority of the load, reducing stress on surrounding bone, leading to bone resorption and implant loosening.^6,7^ Additionally, Ti6Al4V exhibits excellent biocompatibility, minimizing adverse tissue reactions and promoting long-term implant success.^8^ Its corrosion resistance ensures implant durability and prevents the release of harmful particles.^9^ Furthermore, its formability allows for diverse implant designs, catering to various anatomical needs.^10^ However, another significant concern associated with orthopedic implants is infection. Bacterial attachment and subsequent biofilm formation (microcolonies of bacteria encased in a protective matrix) on the implant surface can lead to inflammation, pain, and even implant failure. Reported data suggests that prosthetic joint infections (PJIs) occur in 1–2% of total joint replacement surgeries, significantly impacting patient outcomes and healthcare costs.^11^ To address this challenge, various strategies have been implemented, including antimicrobial coatings and strict aseptic surgical techniques. Our focus here lies on surface modification, which plays a crucial role in reducing opportunistic bacteria attachment and biofilm formation by 50–90%, thereby preventing infection and subsequent implant failure.^12^ It essentially creates a less hospitable environment for bacteria on the implant surface. While creating alloys with inherent antibacterial properties (*e.g.*, titanium–silver alloys) has been explored,^13^ these often compromise the essential mechanical characteristics required for safe and effective implants. This highlights the importance of surface modification for titanium and its alloys, particularly considering their unique properties and mechanical compatibility with bone.

Several surface modification technologies have been implemented to enhance the functionality of implants. Among these, laser surface modification has emerged as a promising technique due to its precision, controllability, and versatility.^14,15^ This technique offers a unique advantage in creating micro and nanoscale features that mimic bone's natural topography, promoting osseointegration, the process of direct contact and bonding between living bone and the implant surface.^16^ Additionally, laser surface modification can be used to create surface structures that disrupt bacterial adhesion and biofilm formation, thereby enhancing the implant's antibacterial properties.^16^ However, previous studies have primarily focused on surface topography modifications without integrating antibacterial compounds into the processed surface. This gap necessitates the development of strategies to incorporate long-lasting and broad-spectrum antibacterial properties into laser-modified surfaces to further combat implant-related infections.

Titanium–silver alloys have emerged as a promising avenue for implant applications, offering broad-spectrum antibacterial properties with lower toxicity compared to alternative materials.^16^ The integration of titanium–silver alloy structures into implants has spurred innovative techniques, particularly through 3D printing using mixed particles of silver and titanium *via* powder bed fusion methods. Song *et al.*^17^ investigated this approach, by utilizing laser powder bed fusion (LPBF) to form Ti–Ag alloys. Impressively, the process showcased excellent antibacterial properties against *Staphylococcus aureus* (*S. aureus*). However, the study raised concerns about the mechanical properties of the 3D printed alloys which showed higher levels of brittleness due to the precipitation of Ti_2_Ag in the 3D printed structures, questioning the practicality of these alloys for implantable applications. In another study Hengel *et al.*^18^ explored an alternative 3D printing approach where they employed selective laser melting (SLM) followed by plasma electrolytic oxidation to introduce silver and zinc nanoparticles into titanium surfaces. While this method holds promise, offering intriguing possibilities, apprehensions persist regarding its mechanical properties' consistency of the 3D printed parts in meeting required standards for implant application. Therefore, many research efforts have shifted towards modifying the surface of titanium alloys with silver compounds to minimize changes in their bulk properties. One notable approach involves laser surface texturing, where the titanium surface is laser textured, followed by post-processing techniques to incorporate silver elements. The work by C. P. Priyanka *et al.*^19^ on Ti–6Al–4V showcased laser texturing followed by coating with TiN and Ag using physical vapor deposition. This method yielded stable surfaces with roughness and hydrophilic characteristics, coupled with robust antibacterial properties against *S. aureus*, exhibiting biocompatibility with HOS cells *in vitro*. In a similar study, Inês M. R. Gonçalves *et al.*^20^ employed laser surface texturing followed by a post-coating with a silver particle solution and sintered onto the surface through heat press. The modified surface demonstrated antibacterial properties and biofilm prevention for up to 72 hours against *P. gingivalis* and *P. intermedia*. Despite the promising antibacterial properties of post-laser surface modification techniques, a critical challenge with such methods is the potential to disrupt and fill in finely textured nano features created by laser surface texturing. Moreover, the materials used for coating often lack strong chemical bonding, leading to potential leaching of antibacterial agents from the surface, limiting their efficacy to only a few days. An alternative research avenue explores concurrent laser texturing and silver alloy formation. Qiao *et al.*^21^ recently investigated this by coating a titanium implant with a slurry of Ag powder and PVA solution, followed by laser texturing. While this process achieved unique alloy layers with antibacterial properties against *Escherichia coli* (*E. coli*) and *S. aureus*, challenges persisted.^22,23^ The relatively thick alloy layer with a high silver content raised concerns about overall mechanical interface, biocompatibility, and bone cell mineralization, aspects not thoroughly explored in the study. The focus on sintering an alloy formation at the interface somewhat neglected the creation of a nano-textured surface for enhanced bone cell attachment.

**Fig. 1:**
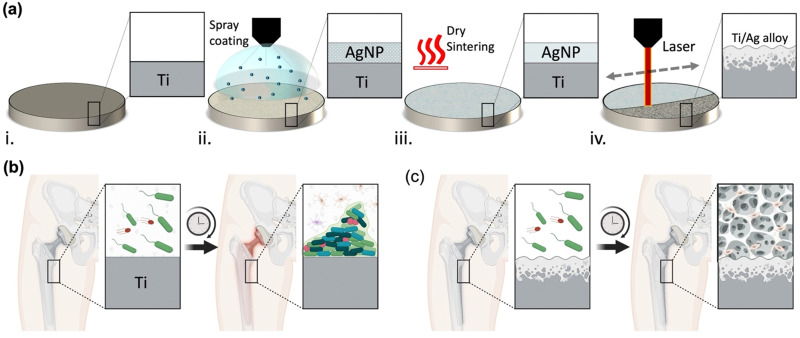
(a) Schematic illustration showing the step-by-step process of laser-assisted surface alloying (LSA) to modify orthopedic implant surfaces with TiAg alloy for enhanced antibacterial and osteoinduction properties. (b) Schematic orthopedic implant without surface modification which are of high risk of infection and implant failure. (c) Schematic of orthopedic implant modified with LSA-TiAg alloy that imparts long-lasting antibacterial properties and enhanced osteoinduction properties to the orthopedic implant surface.

In light of these challenges and findings, there is a pressing need for laser surface modification technologies capable of creating nano-textured alloy surfaces with minimal impact on overall mechanical properties at the bone–implant interface. The ideal modification should manifest as a thin, nano-textured alloy interface with stable hydrophilic characteristics, allowing for bone cell attachment, while maintaining a stable silver content within the alloy structure.^24^ This imperative is particularly critical for implantable applications demanding enduring antibacterial characteristics for periods extending beyond 10 days. To address this need, we demonstrate for the first time the utilization of selective laser-assisted surface alloying (LSA) and nanotexturing of Ti6Al4V implant surfaces with silver nanoparticles (AgNPs) to enhance both antibacterial and bone mineralization properties without changing the bulk characteristics of the metal while preserving minimal cytotoxicity. Our process involves a rapid and unique two-step spray coating of AgNP followed by a selective laser-assisted surface alloying processing Ti6Al4V implant surface as illustrated in Fig. 1a. The local heat generated by the laser process allows the unique and simultaneous intermetallic alloying and texturing of the implant surface with enhanced surface roughness that is critical for promoting bone mineralization.^25^ The antibacterial and osteointegration properties of the LSA–TiAg can help to overcome the race between bacteria and bone cells in orthopedic implants, Fig. 1b.^26^ By modifying the surface of the implant, the antibacterial properties can prevent bacterial colonization, while the enhanced osteointegration properties can promote bone cell adhesion and colonization, Fig. 1c. The nano-textured titanium-silver alloy surface, resulting from the laser concurrent alloying and texturing exhibits robust antibacterial properties through multifaceted mechanisms. Silver ions (Ag^+^) released from surface directly disrupt bacterial cell membranes, generate reactive oxygen species (ROS), and inhibit vital enzymes, collectively leading to bacterial death.^27–29^ Simultaneously, the laser-induced textured features reduce bacterial adhesion, impeding biofilm formation, and increase the surface area, facilitating enhanced interaction between the surface and bacteria. This synergistic interplay, reinforced by the sustained superhydrophilicity of the surface, underscores its profound and lasting antibacterial efficacy (see Fig. S1 in ESI†). This creates an environment that is favorable for bone growth and healing, while simultaneously inhibiting the growth of harmful bacteria that can cause infection and implant failure. Ultimately, this approach can help to improve the long-term success of orthopedic implants and enhance patient outcomes. A systematic investigation with varying laser processing power was performed to assess the overall surface morphology, chemical composition, and mechanical properties of the processed Ti6Al4V implant surface. Furthermore, long-term antibacterial properties of the developed surface with varying processing conditions were tested against a model Gram-positive (*S. aureus*) and Gram-negative (*E. coli*) bacteria. Finally, the *in vitro* bone mineralization assay of the developed implant surfaces with different laser powers were tested using Mesenchymal Stem Cells (MSC), and the calcified extracellular matrix levels were estimated using alizarin red staining.

## 2. Experimental section

### 2.1. Laser-assisted surface alloying process

Initially, all Ti6Al4V circular coupons with diameter of 1.2 cm were subjected to a plasma treatment process for surface cleaning.^30,31^ The plasma-treated Ti surfaces were spray-coated with AgNPs ink (JS-A101A Silver Nanoparticle Ink, Novacentrix, Austin, TX, USA) with an average particle size of 40 nm and final loading ratio of 3.57 wt%. To obtain a homogenous coating of AgNPs on the surface of the Ti circular coupons, a conformal spray coating system (Nordson, Q6800) was used. The weight to area ratio of coated AgNP film on Ti alloy surface was estimated to be 8.4 g m^−2^. The coated samples were then dried and sintered on a hotplate of 100 °C for 10 s. Next, laser surface treatment with different processing powers (0, 8, 16, 24, 32, and 40 W) and a fixed scanning speed of 0.4 m s^−1^ was applied on AgNPs coated Ti samples. All laser processes were performed with a computer-controlled Nd:YAG fiber laser system (PLS6MW, Universal Lasers, Inc., Scottsdale, AZ) with pulse duration in the nanosecond range set at raster scanning mode. The spot size and wavelength of the operating laser beam were 25 μm and 1.06 μm, respectively. Hereafter, the “Ti” is referred to pristine Ti6Al4V sample, “AgNP–Ti” for the AgNP-coated Ti without laser surface treatment, and LSA-TiAg refers to laser processed AgNP–Ti samples with various laser processing powers that are mentioned in our manuscript as: 8 W LSA-TiAg, 16 W LSA-TiAg, 24 W LSA-TiAg, 32 W LSA-TiAg, 40 W LSA-TiAg.

### 2.2. Material characterization

In order to systematically investigate the effect of laser process on the effective AgNP–Ti alloy formation on the processed samples, a series of surface chemical and microstructure analysis was performed. The field emission scanning electron microscope (FE-SEM) (Hitachi-S4800) with built-in energy-dispersive X-ray spectroscopy (EDX) was used to understand the morphology of Ti samples before and after the LSA process on Ag-coated samples.^32^ Additionally, the cross-sectional imaging of the developed surface was analyzed using Focused Ion Beam (FIB) milling (Helios 5 DualBeam, Thermofisher Scientific) and EDX analysis at the interface. The elemental composition of Ti samples before and after LSA process was determined by EDX and grazing angle X-ray diffraction (GIXRD, PANalytical Empyrean Diffractometer system with a Cu Kα1, *λ* = 1.5406 Å source) analysis. The adhesion strength of the Ag coating on Ti was assessed using a scotch tape peel test methodology. In this procedure, 25 mm × 25 mm sections of 3M adhesive tape were affixed to the Ag-coated Ti samples. The tape was subsequently peeled off at a 90° angle, maintaining a consistent rate throughout the process. The analysis involved visually inspecting the adhesive surface on the tape to quantify the extent of material detachment from the coated surface. Water contact angle (WCA) measurements were performed using Advanced Goniometer (290f1, ramé-hart Instrument Corporation) to understand the surface wettability of laser processed and unprocessed Ti samples. To study the mechanical properties of the samples, microhardness tests were performed using a Rockwell hardness tester (SPI Rockwell Type Hardness Tester) with 120° diamond indenter tip and Rockwell C test blocks. All measurements were performed in three replicates and the average and standard deviation were recorded.

### 2.3. Antimicrobial assay

The long-term antibacterial characteristics of different specimens including Ti, AgNP–Ti, 8 W LSA-TiAg, 16 W LSA-TiAg, 24 W LSA-TiAg, 32 W LSA-TiAg, and 40 W LSA-TiAg were analyzed by placing each specimen in 2 mL of PBS solution for duration of 0, 48, 96, 144, 192, 240, and 288 h. At each test point, the test samples were removed, washed with 70% isopropanol (IPA) three times, and dried under a nitrogen atmosphere for 10 min. Next, at each time point the antibacterial activity of the surface was tested using the contact-killing method against both Gram-negative *E. coli* (ATCC 25922) and Gram-positive *S. aureus* (ATCC 25923) bacteria. For this test, a 50 μL bacterial suspension containing 7 log_10_ CFU mL^−1^ was aliquoted onto the test samples and incubated for 24 hours at 37 °C in a 24-well cell culture plate. After the incubation period, the samples were aspirated multiple times with a 100 μL of PBS (supplemented with 0.1% Tween-20 (Sigma-Aldrich)) to detach the bacteria on the surface. Next, a 20 μL suspension was collected from the sample surfaces and subsequently serially diluted. The diluted suspension was plated onto TSB agar plates and incubated at 37 °C for 16 hours to determine the number of colony-forming units (CFU).^33–36^ All experiments were performed in triplicate to ensure appropriate statistical variations. Further, to assess the extent of the bacterial cell wall damage caused by each surface, a LIVE/DEAD BacLight kit (Thermofisher) was examined on *E. coli* as a representative bacterial strain. For this test, 50 μL of 7 log_10_ CFU mL^−1^ of bacterial suspension was placed on each sample and incubated at 37 °C for 24 h. After the incubation period, the bacterial samples from the surfaces were recovered and subjected to live/dead staining analysis. To perform the analysis, 100 μL of the bacterial samples were mixed with 3 μL of the dye mixture (containing equal volumes of SYTO 9 and propidium iodide) and incubated in the dark for 15 minutes. The bacterial cells were imaged using an inverted epi-fluorescence microscope (Nikon Eclipse TS2 Olympus, Waltham, MA) equipped with a photometric cool snap dyno camera under a 40× objective and 10× optical lens using NIS-Elements D software.^37^ All experiments were performed in triplicate to ensure appropriate statistical variation.

### 2.4. Cell mineralization study

To examine the cell adhesion and osteointegration characteristics of developed LSA surfaces, *in vitro* cell mineralization studies were performed. For this, mesenchymal stem cells (MSCs) media (ScienCell) was utilized to culture the cells prior to treating the surfaces. Freshly prepared complete media (Gibco DMEM/F-12 media with 10% fetal bovine serum (FBS) supplied with 1% penicillin and streptomycin) was used for all cell culture experiments.^33,38–40^ Cells between passages 3 and 5 were used for this experiment. Confluency of the cultured cells was observed under the microscope and the cells were trypsinized and suspended back to the DMEM/F-12 media and the concentration was measured using a countess cell counter (Countless, Invitrogen). All test samples were placed in 24 well tissue culture plates followed by the addition of 2 mL cell suspension with a cell seeding density of 2 × 10^4^ cells per cm^2^ and placed in an incubation chamber at 37 °C. The growth medium was exchanged with the differentiation media after 24 h and this step was repeated every 48 h with 1 mL of fresh media till day 7. After that, the differentiation media was changed every 24 h for another 14 days to continue the bone mineralization process. After three weeks, the test specimens were fixed with 2% glutaraldehyde solution for 1 h and rinsed three times with DI water. Further, 500 μL of Alizarin red staining (Sigma-Aldrich) solution was added into each well with test specimens and subjected to rocking agitation for 1 h. The stained specimens were rinsed and were allowed to sit in DI water overnight under dark conditions. The monolayers formed on the surface of specimens were removed through tweezers and examined using an inverted microscope (Nikon Eclipse TS2 Olympus, Waltham, MA) equipped with a monochrome camera (photometric cool snap dyno camera) and NIS-Element's imaging software. For quantitative analysis of cell mineralization, monolayers obtained from the surface of each specimen were detained using 200 μL hydrochloric acid (2 M) at 85 °C for 2 h in a heat block. The obtained debris of the monolayer was centrifuged for 10 min at 20 000 rpm, and the solubilized Alizarin Red was quantified by recording the absorbance of each sample at 405 nm wavelength using a spectrophotometer (Versa Max, Molecular Devices) immediately after neutralization with 6 M NaOH solution. The mineralization percentage on each surface was calculated using the following equation:
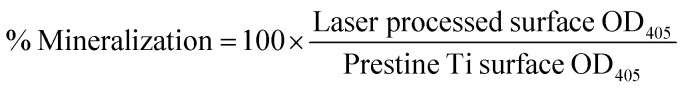
where the OD_405_ is the optical density value measured for each sample at 405 nm wavelength.

### 2.5. Statistical analysis

All material characterization analysis such as WCA, hardness, elemental composition analysis was repeated 3 times on to derive the average and standard deviation values. All collected antibacterial and cell mineralization data were analyzed by one-way analysis of variance (ANOVA) followed by Tukey's multiple comparison *post hoc* test to determine any statistical significance of difference between the test groups. The level of statistical significance was set at *p* ≤ 0.05.

## 3. Results and discussion

### 3.1. Microstructure characterization

**Fig. 2:**
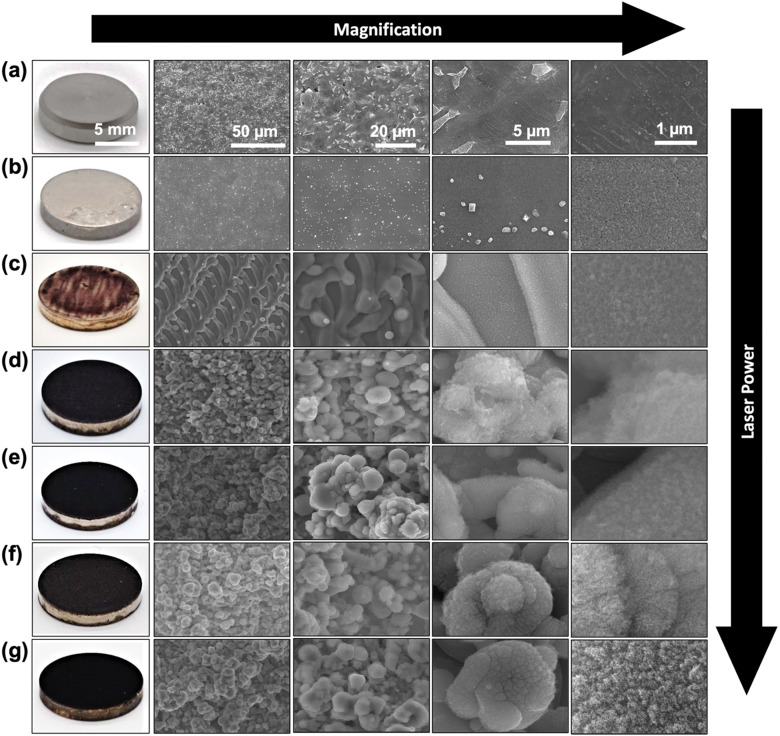
Surface photos and SEM micrographs of (a) Ti, (b) AgNP–Ti, (c) 8 W LSA-TiAg, (d) 16 W LSA-TiAg, (e) 24 W LSA-TiAg, (f) 32 W LSA-TiAg, and (g) 40 W LSA-TiAg samples at different magnifications.

The effect of laser processing power on microstructure and surface morphology of Ti specimens was evaluated using SEM imaging. As shown in Fig. 2a, the pristine Ti specimen (marked as Ti) with grayish look in realistic image, shows a smooth and featureless metallic surface. The high magnification SEM images reveal a fine network of interconnected grain boundaries of the Ti sample and some micrometric bumps and defects. The SEM images of the spray-coated AgNP–Ti without the laser surface treatment showed a shiny silverish appearance with an average coating thickness of 937.6 nm as observed from cross-section SEM images (shown in Fig. S2 of ESI†). Low magnification SEM images from these samples also showed a homogeneous topography of the AgNPs on the Ti surface. The high magnification SEM images reveal the fine details of AgNPs on the Ti surface where the evenly distributed AgNPs were to some extent fused to each other by the thermal annealing process (Fig. 2b). The Ag coating also should a strong adhesion to the Ti surface which was confirmed with no discernible signs of material peeling off by the scotch tape test (as shown in Fig. S3 of ESI†). On the other hand, the LSA surfaces demonstrated dark appearance in the photos and rough topography with developed hierarchical micro/nano structures in their SEM images (Fig. 2b–g). The color change of the LSA specimens can be attributed to the increased light scattering from the developed micro/nano features. The SEM images from the top view of the 8 W LSA-TiAg samples demonstrate the formation of micron-scale groove (line periods of 25 μm), which are attributed to the scanning pattern of the laser beam during the surface treatment process. In contrast, the samples processed with higher laser processing power conditions (>16 W) exhibited a noticeably darker black color in the photos (Fig. 2d–g). Furthermore, low magnification SEM images of these samples showed more uniform formation of hierarchical porous microstructure with microspheres averaging around 5 μm in diameter with a well-defined shape and size distribution. High magnification SEM images from the LSA samples show the presence of cauliflower-like (5–10 nm) nanostructures uniformly covering the entire surface of the processed sample. This consistent morphology was observed throughout the samples and was independent of the laser processing power condition.

### 3.2. Elemental analysis

**Fig. 3:**
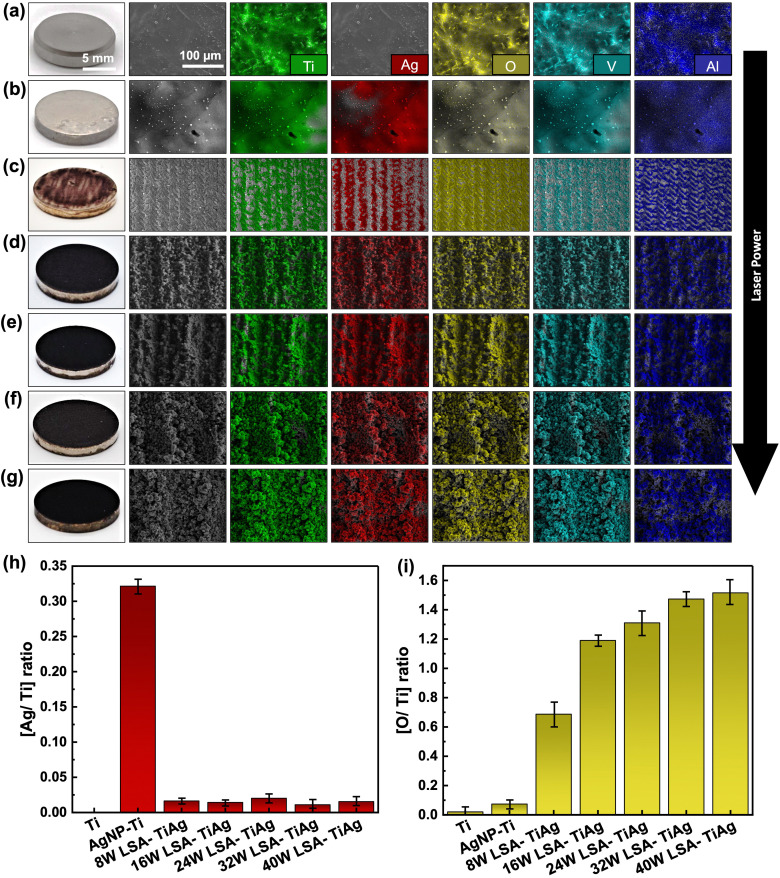
EDX elemental maps of Ti, Ag, O, V, and Al on the surface of (a) Ti, (b) AgNP–Ti, (c) 8 W LSA-TiAg, (d) 16 W LSA-TiAg, (e) 24 W LSA-TiAg, (f) 32 W LSA-TiAg, and (g) 40 W LSA-TiAg samples. Relative changes in the level of elemental (h) silver and (i) oxygen on the samples surface.

The elemental composition of the pristine Ti6Al4V and LSA specimens was analyzed using energy-dispersive X-ray spectroscopy (EDX) mapping of the surfaces (Fig. 3). The results showed that the primary elements of Ti, Al, and V are present in all specimens as depicted in Fig. 3(a–g), which aligns with the established literature describing the elemental composition of the Ti6Al4V alloys. Evidently, the pristine sample did not show any Ag content while demonstrating the presence of oxygen that can assign to the native oxide layer on the Ti6Al4V specimen (Fig. 3a). As expected, the Ag NP spray coated Ti6Al4V samples without the laser treatment showed a significant increase in the Ag content. Meanwhile the EDX map for oxygen has noticeably faded due to the AgNP coating covering the native titanium dioxide layer on the underlying Ti6Al4V specimen (Fig. 3b). Similar to the previous SEM images, the EDX elemental map also showed the formation of micron-scale grooves on the surface of specimens that were processed with an operating power of 8 W. This phenomenon is attributed to the scanning motion of the laser beam over the processed sample. As evident in the parallel lines regions that the laser has scanned over the surface, there was a noticeable removal of silver content, and an increase in titanium and oxygen level with a higher contrast in the color of the EDX map. This can be explained by the removal of the silver coating within the ablated regions, exposing the underlying titanium substrate. Also, inducing thermal oxidation and the formation of titanium dioxide within the ablated regions due to the high-temperature laser energy and the presence of oxygen in the ambient environment resulted in a higher content of oxygen within the laser-ablated regions. In contrast, samples processed with higher laser powers (>16 W) not only showed deeper and rougher textured surfaces, but also demonstrated a more uniform distribution of all elements, including Ag, Ti, and O throughout the processed samples, both inside and outside the micron scale grooves formed through laser processing. This phenomenon can be explained by the more violent interaction of the higher laser energy with the heat-affected zone, resulting in an effective intermixing of the coated AgNP with the underlying Ti substrate through the processes of simultaneous vaporization and sublimation, Fig. 3c–g.

Fig. 3h and i provide a summary plot of the changes in the Ag/Ti and O/Ti ratios obtained from the EDX analysis. The plot reveals that the laser ablated samples had a similar overall low Ag content on the surface after the laser process, as indicated by the Ag/Ti ratio (∼0.02). However, the O/Ti ratios showed a significant increase of 1.22 as the laser power was increased above 16W and had a relatively stable level after this laser power processing condition. This increase can be attributed to the higher content of metal oxidation due to the elevated thermal energy density provided by the laser beam and the presence of oxygen in the ambient, resulting in the formation of more metal oxide constituents on the processed surfaces also referred to laser-induced oxide.^41–43^

### 3.3. Crystallographic study

**Fig. 4:**
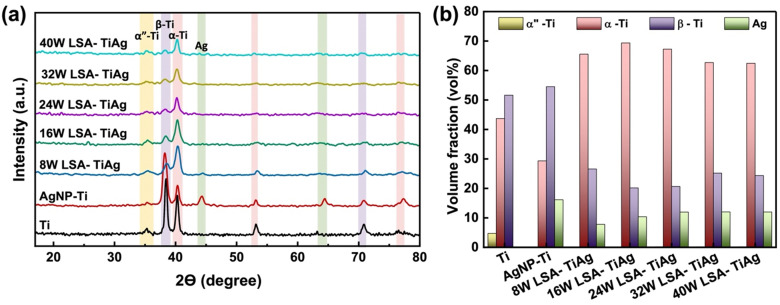
(a) GIXRD spectra of pristine Ti, AgNP–Ti, and LSA TiAg samples. (b) Volume fraction changes of the different crystalline phases in the specimens with different processing conditions.

To confirm the effective alloying of Ag NPs into the Ti specimen's surface with varying laser powers, we performed crystal analysis using GI-XRD. Fig. 4a shows the GI-XRD pattern of the pristine Ti, AgNP–Ti, and LSA samples. The pristine Ti had a small level of martensite *α*′′ and high intensity for α-Ti and β-Ti phases, which appeared at 2*θ* values of 35.3 (*α*′′), 40.3, 53.2, 76.5 (*α*), and 38.5, 70.8 (*β*). The volume percentage of β-Ti (JCPDS file #44-1288), which is attributed to body-centered cubic (BCC) Ti phase, was higher than α-Ti (JCPDS file #44-1294), a hexagonal closed pack (HCP) Ti phase. In contrast, AgNP–Ti samples showed new peaks at 44° and 64°, confirming the presence of face-centered cubic (FCC) Ag crystalline structure. Based on the area under the peaks, we calculated the total volume percentage of each phase, and plotted them in Fig. 4b. Samples with AgNP coating and no laser surface treatment had an increase in the volume percentage of Ag, a consistent volume ratio of β-Ti, and a 14% decrease in α-Ti. In contrast, laser surface treatment resulted in a clear decrease in the volume percentage of Ag. This could be attributed to factors such as Ag being vaporized by the laser beam, and a portion of it being alloyed with the underlying Ti substrate during the surface alloying process.

Notably, laser-processed samples with higher than 16W showed a relatively consistent volume percentage of Ag (12%) with a dramatic increase in α-Ti phase (by 36.2%) and a decrease in the β-Ti phase (by 27.9%), with a gradual shift in peak position of 5° by increasing the laser processing power. Previous studies have shown that laser surface treatment leads to a rapid temperature rise and cooling which can result in the predominant formation of α-Ti phase of a thin layer on the surface, a brittle layer consisting of α-phase Ti with an oxygen-rich content.^44,45^

However, our results demonstrate that Ti6Al4V after AgNP coating followed by laser surface treatment contain a high percentage of β-Ti phase and Ag, which are associated with TiAg alloy formation, despite the presence of α-phase Ti. These findings further confirm that our process provides a unique approach to selectively form TiAg alloy with a high content of oxide compounds without necessitating a change in the bulk properties of the Ti6Al4V processed material.

### 3.4. Surface activity analysis

**Fig. 5:**
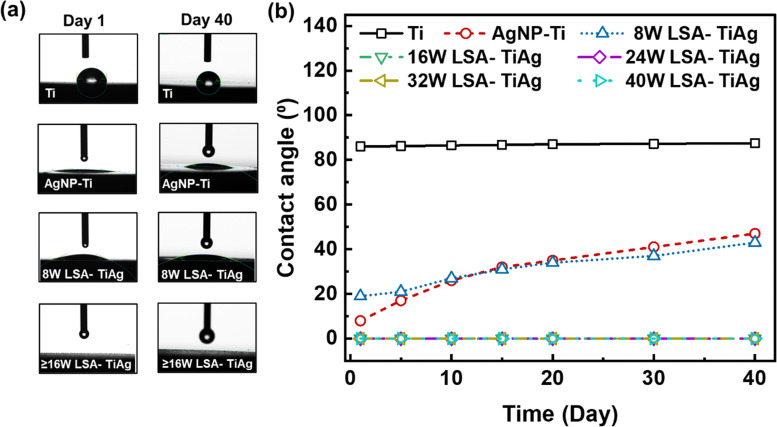
(a) Water droplet on the surface of pristine Ti, AgNP–Ti, and various LSA Ti–Ag samples on day 1 and after 40 days of study. (b) Change in the water-contact angle on different surfaces as a function of time.

To assess the long-term wettability and hydrophilicity characteristics of pristine Ti, AgNP–Ti, and LSA TiAg surfaces, water contact angle (WCA) measurements were conducted on untreated and surface-treated Ti specimens over a period of 40 days. Fig. 5 displays recorded water droplet images and comparative WCA data for different samples. The WCA value is an important characteristic to determine the surface activity and cell interaction with the surface of orthopedic implants. As shown in Fig. 5a, the water droplet on pristine Ti sample formed a complete sphere with an average WCA of 84° and remained stable over 40 days (Fig. 5b). In contrast, the AgNP–Ti surfaces without laser surface treatment showed a significant decrease in WCA value to approximately 20°, indicating higher surface energy due to the presence of AgNPs on the surface. However, the WCA gradually increased over time, which could be due to the unstable surface properties of the AgNP. The LSA-TiAg surfaces with low laser powers of 8 W exhibited an initial WCA value and a gradual increase over time, similar to the AgNP–Ti surfaces without laser treatment. Conversely, LSA specimens with high laser processing powers (>16 W) demonstrated a stable super hydrophilic behavior (0° WCA) for 40 days.

It is imperative to note that the laser surface modification process is carried out in ambient conditions, where the localized heat generated not only induces intermixing and alloy formation of titanium and silver but also triggers the partial oxidation of both metals due to the ambient oxygen presence. The resulting metal oxides, such as titanium oxide (TiO_2_) and silver oxide (Ag_2_O), are renowned for their intrinsic hydrophilicity. This property stems from the presence of polar hydroxyl (OH) groups on their surfaces, which readily form hydrogen bonds with water molecules.^46^ This, in turn, promotes water droplet spreading and leads to lower WCAs.^47^ To delve into the observed superhydrophilic behavior, we turn to the Wenzel force theory, a fundamental framework developed by Robert N. Wenzel in 1936, elucidating the relationship between surface roughness and wetting behavior. On a smooth surface, the liquid droplet sits on a well-defined area, with the contact angle reflecting the inherent properties of the liquid and the solid surface.^48,49^ As a surface becomes rough, as is the case with the laser-textured surfaces in this study, the liquid droplet interacts with a larger surface area due to the peaks and valleys of the roughness.

This interaction leads to two scenarios: For hydrophobic materials, roughness amplifies the water-repelling effect, causing the droplet to bead up more tightly and resulting in a higher contact angle compared to a smooth surface. Conversely, for hydrophilic materials, the roughness enhances the interaction with water, allowing the droplet to spread more readily and leading to a lower contact angle compared to a smooth surface. In the case of our study, the laser-textured surfaces create hierarchical micro and nanoscale roughness, as confirmed by SEM analysis. When these rough features are further covered with hydrophilic metal oxides, the overall surface interaction with water becomes dominated by the combined effect of increased surface area and the inherent hydrophilicity of the oxides. This synergy results in super hydrophilic behavior, where the water droplet instantaneously wicks into the porous matrix of the laser-processed surface. The observed stability of superhydrophilicity over time, particularly for surfaces treated with higher laser power, can be attributed to the extent of metal oxide formation and the characteristics of surface roughness. Higher laser processing power results in more oxidation of the surface, resulting in a greater abundance of hydrophilic metal oxides and a stronger super hydrophilic effect. Additionally, higher power settings create a more intricate and pronounced roughness, further enhancing the combined effect of roughness and the presence of hydrophilic metal oxides on superhydrophilicity. These results demonstrate the potential applicability of this process for orthopedic implants where a stable hydrophilic surface is critical for promoting cell attachment, mineralization, and reducing the probability of implant rejection.^50^

### 3.5. Mechanical hardness assessment

**Fig. 6:**
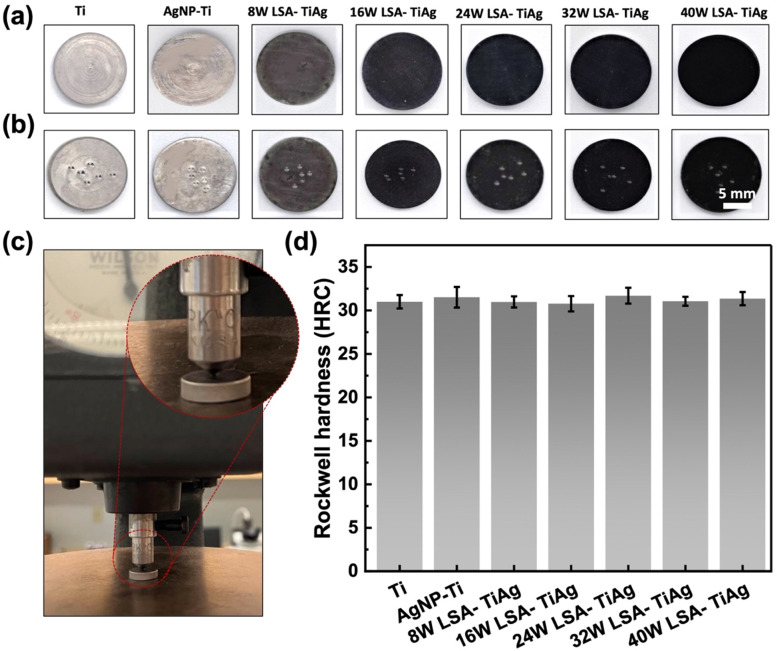
Surface images of pristine Ti, AgNP–Ti, and LSA Ti–Ag samples (a) before hardness test and (b) after hardness test. (c) Rockwell C hardness test setup and (d) Rockwell C hardness measurement of samples with different processing conditions.

The mechanical properties of the implant surface must be similar to those of the host bone to avoid stress shielding complications. Thus, an essential consideration for the LSA surface modification process is to ensure that it does not alter the bulk mechanical properties of the underlying Ti implant. In order to evaluate the effect of laser assisted alloying, the hardness of the pristine and LSA samples was investigated by studying the Rockwell C hardness of the specimens. Fig. 6a–c shows the surface of the samples before and after the Rockwell C hardness test. The images clearly show mechanical indents created by the indenter throughout different areas of the specimen surface. Fig. 6c shows the instrument used for the test, with an inset of a zoomed-in image of the indenter tip. As demonstrated in Fig. 6d, the Rockwell hardness values of all the samples were constant, with an average value of 31 ± 0.6 HRC. These values were quite similar to those of the control pristine Ti samples. This observation revealed that the LSA process is a highly superficial surface modification that does not change the bulk mechanical properties of the processed Ti implant.

### 3.6. Antibacterial analysis

**Fig. 7:**
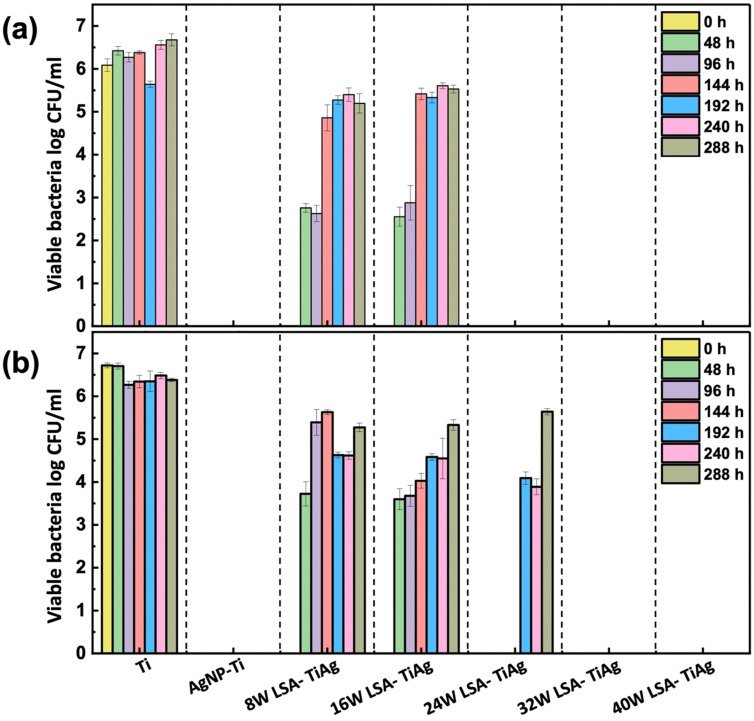
Long-lasting antibacterial efficiency assessment of pristine Ti, AgNP–Ti, and LSA Ti–Ag samples against (a) *E. coli* and (b) *S. aureus*.

The interstitial alloys were tested for their antimicrobial properties against two common implant-based infection bacteria, *E. coli* and *S. aureus*, with a CFU concentration of 10^7^ log_10_ mL^−1^. A facile contact killing method was used to evaluate the static antibacterial effects of the interstitial alloys, which included Ti, AgNP–Ti, 8 W LSA-TiAg, 16 W LSA-TiAg, 24 W LSA-TiAg, 32 W LSA-TiAg, and 40 W LSA-TiAg. The results showed that the AgNP–Ti surface had strong antimicrobial properties compared to pristine Ti, which showed no discernible change in the bacterial population. The long-lasting bactericidal properties of AgNP can be attributed to its antimicrobial mechanism of action, which causes cell membrane damage and intra-cellular disruption in the bacteria, Fig. 7.^51^ However, the laser surface treatment showed a noticeable change in the repeated antibacterial tests over time.

Samples processed with lower laser processing conditions (<16 W) exhibited a decrease in overall bactericidal properties after a 3-log kill-off against both *E. coli* and *S. aureus* within 96 h and completely lost their bactericidal properties beyond this point. In contrast, LSA-TiAg samples with higher processing power (>16 W) exhibited sustained bactericidal properties over the course of 288 h study. Results showed that a minimum laser processing power of 24 W was required for stable antibacterial properties against *E. coli* (*p* < 0.05), while a minimum processing power of 32 W was required for stable antibacterial properties against *S. aureus* (*p* < 0.05).

These discrepancies in the duration of antibacterial efficacy under different laser processing power conditions can be attributed to multiple factors. Firstly, the variance in bacterial cell wall structure plays a pivotal role; Gram-positive bacteria, such as *S. aureus*, possess a thicker peptidoglycan layer compared to Gram-negative bacteria like *E. coli*, acting as a physical barrier that hinders the penetration of silver ions and other antimicrobial agents.^52^ Additionally, at higher laser power settings (32 W), there is improved intermixing of silver with the titanium substrate, resulting in an alloy with a slightly higher silver content, as observed in the surface topography and elemental analysis. This elevated silver content, coupled with increased surface roughness and effective surface area provided by the nanotextured features at 32 W, contributes to the more sustained antibacterial effect observed against *S. aureus* (up to 288 h). The interplay of bacterial cell wall structure, silver content, and surface topography elucidates the observed differences in laser power requirements for sustained antibacterial activity against *E. coli* and *S. aureus*. As detailed in the manuscript, a minimal laser power of 32 W is necessary to impart the essential surface characteristics for long-lasting antibacterial properties against both *E. coli* and *S. aureus* for up to 288 h.

### 3.7. Biomineralization assessment

**Fig. 8:**
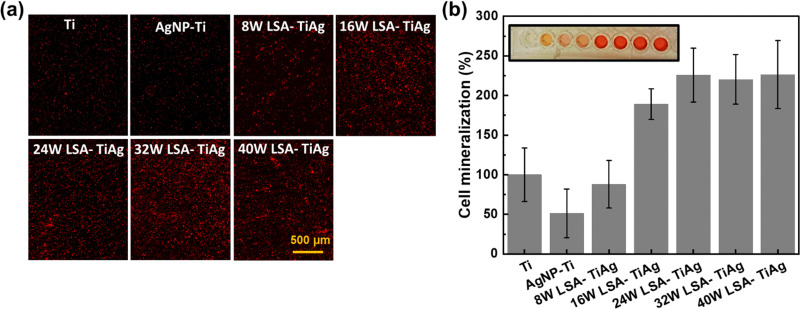
(a) Microscopic images of the alizarin red stained surfaces of samples showing cell-mineralization efficiency on different surfaces. (b) Quantified level of mineralization on pristine Ti, AgNP–Ti, and LSA Ti–Ag samples prepared with different laser processing powers.

The effect of different LSA conditions on cell mineralization properties was studied to assess osteointegration on the developed surface. Primary MSC cell lines were used for this study as they are in the predifferentiated proliferative state that enhances calcification. In this study, the osteoinductive properties of the LSA-TiAg implant surfaces were studied by eliminating all the osteogenic induction factors, and the level of calcium deposits on the processed surfaces was used as a marker of osteoinductive properties of the surface, which was quantified by staining the surface using an inorganic alizarin red dye. Fig. 8a shows the microscopic images of the alizarin red stained surfaces of different samples. As observed, the AgNP–Ti surfaces showed a significant decrease in cell mineralization due to the toxicity of AgNP to the MSC cells, leading to decreased proliferation and mineralization. In contrast, LSA-TiAg surfaces showed a noticeable increase in overall cell mineralization, which can be attributed to the high surface roughness and enhanced surface wettability of the laser-treated sample, promoting more surface area and anchoring sites for cellular attachment and mineralization. Further, the level of mineralization was quantified by recording the optical absorbance of each sample at 405 nm wavelength, as shown in Fig. 8b. The results showed that the AgNP–Ti surfaces had a 62% decrease in cell mineralization percentage.^53^ However, after laser surface treatment, the level of mineralization increased significantly (220%). LSA-TiAg samples processed with laser processing powers above 24 W had relatively stable and similar bone osteoinduction properties.

This observation can be attributed to several key factors. Firstly, higher laser powers promote efficient intermixing of silver with the titanium substrate, leading to effective immobilization and the formation of a silver–titanium alloy. This ensures a consistent and controlled release of silver ions without compromising biocompatibility, maintaining favorable conditions for bone cell attachment and growth. Additionally, these higher power settings result in the formation of metal oxide compounds on the surface, contributing to long-lasting superhydrophilicity, essential for proper bone cell attachment. This sustained hydrophilicity likely plays a crucial role in maintaining stable osteoinduction properties. Furthermore, while higher power settings create deeper laser-etched trenches, the overall cauliflower-like nanofeatures remain consistent and uniformly distributed. These features likely provide sufficient surface roughness and complexity for effective bone cell interaction, contributing to the observed stability in osteoinduction. In summary, the combination of effective silver immobilization, sustained hydrophilicity, and minimal impact on crucial nanofeatures likely explains the stable bone osteoinduction properties observed in samples processed with laser powers above 24 W. The combination of antibacterial properties and bone cell mineralization assessment suggests that LSA-TiAg with a minimal processing power of 32 W exhibits long-term antibacterial and optimal bone mineralization properties, making it particularly suitable for implant-based applications.

A summary of various strategies for the development of laser-enabled antibacterial silver composites on implant surfaces, reported in recent literatures are tabulated and compared in Table S1 provided in the ESI.† Though, these previously reported techniques show a reasonable antibacterial and cell adhesion properties, the long-term performance of the antibacterial effect of the surface while maintaining minimal toxicity is a major concern for effective osteointegration. In this context, the developed LSA-TiAg surface provides a sustained antibacterial for 12 days with a two-fold increase in the osteointegration properties without altering the bulk properties of the implant surface. Further investigations into *in-vivo* performance and long-term durability are imperative to fully ascertain the clinical potential of this groundbreaking technology.

## 4. Conclusion

This study has successfully pioneered a laser-assisted surface modification of titanium implants, incorporating concurrent nanotexturing and alloying with silver, to achieve remarkable antibacterial and osteoconductive properties. Employing optimized laser processing conditions at 32 W and above, we achieved the formation of hierarchical mesoporous structures, featuring cauliflower-like nanostructures (5–10 nm) uniformly distributed over micron-scale lines (25 μm spacing). This resulted in a nano-textured titanium–silver alloy through localized heat-driven mixing, ensuring sustained silver ion release for potential long-term antibacterial efficacy. The ambient processing introduced a metal oxide layer, fostering sustained superhydrophilicity (up to 40 days) and improving biocompatibility. This multifaceted approach demonstrated effective antibacterial activity against both *E. coli* and *S. aureus*, common bacterial strains associated with implant infections, with sustained effects lasting up to 12 days. Remarkably, samples processed with laser powers above 32 W exhibited stable two-fold increase in osteointegration property as compared to the pristine Ti surface. This stability is attributed to factors such as effective silver immobilization, sustained hydrophilicity, and minimal impact on crucial nanofeatures. The Rockwell hardness test confirmed that the laser process had only superficially modified the surface, without affecting the bulk mechanical properties of the processed titanium. This innovative surface modification strategy presents a compelling avenue for developing implants with enduring antibacterial properties and optimal bone osseointegration, potentially mitigating infection risks and fostering successful implant integration within the body.

## Conflicts of interest

There are no conflicts to declare.

## Supplementary Material

TB-012-D3TB02481D-s001
