# Supplementary material for: Laser-assisted surface alloying of titanium with silver to enhance antibacterial and bone-cell mineralization properties of orthopedic implants
Source: J Mater Chem B. 2024 Apr 22;12(18):4489–501. doi: 10.1039/d3tb02481d (PMC11078329; doi:10.1039/d3tb02481d)
Supplement: TB-012-D3TB02481D-s001 [file TB-012-D3TB02481D-s001.pdf]

Electronic Supplementary Material (ESI) for Journal of Materials Chemistry B.
This journal is © The Royal Society of Chemistry 2024

Supporting Information

Sotoudeh Sedaghat[a,c], Akshay Krishnakumar[b,c], Vidhya Selvamani[a,c], James P. Barnard[a], Sina Nejati[a,c], Haiyan Wang [a], David A. Detwiler[b,d], Mohamed N. Seleem[e], and Rahim Rahimi[a,b,c*]

[a]*School of Materials Engineering, Purdue University, West Lafayette, IN 47907, USA*

[b]*Birck Nanotechnology Center, Purdue University, West Lafayette, IN 47907, USA*

[c]*School of Electrical and Computer Engineering, Purdue University, West Lafayette, IN 47907, USA*

[d]*Nanovis, West Lafayette, West Lafayette, IN 47907, USA*

[e]*Department of Biomedical Sciences and Pathobiology, Virginia Polytechnic Institute and State University, Blacksburg, VA, 24061, USA*

*** Email:** rrahimi@purdue.edu

Figures

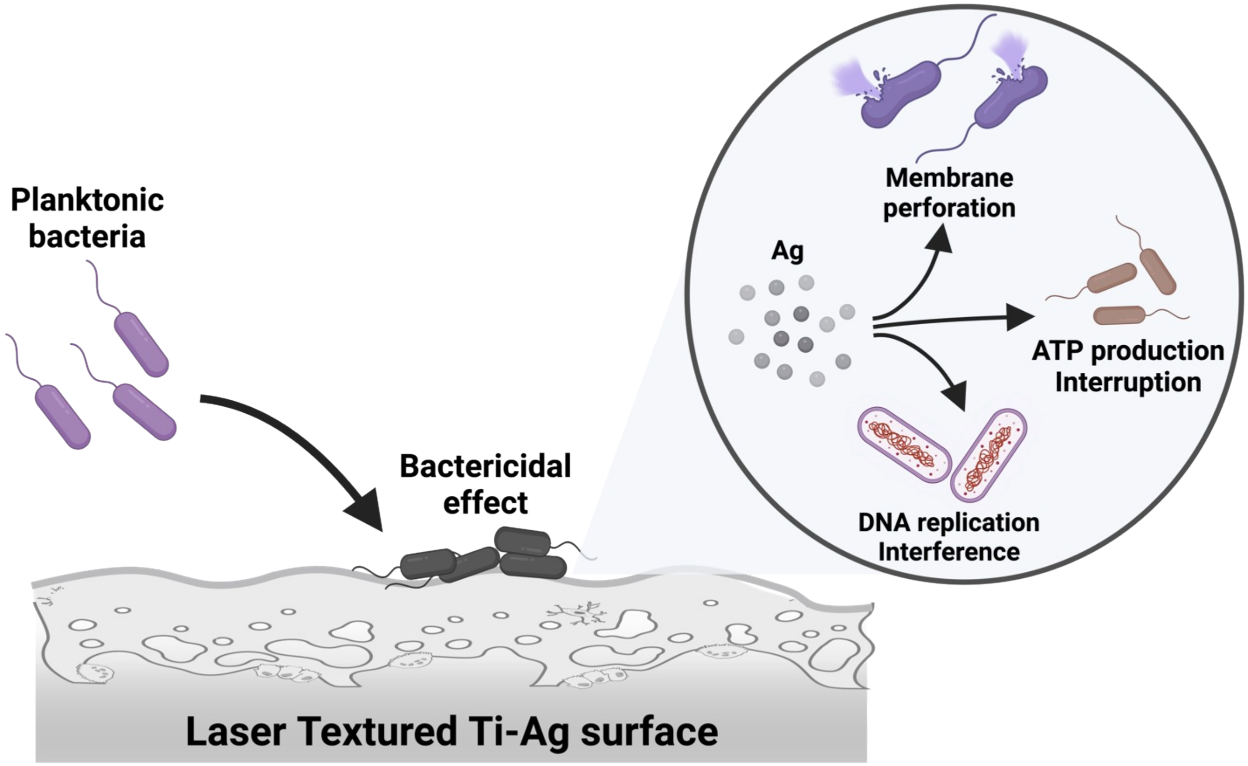

Figure S1. Antimicrobial mechanism of silver in the laser-modified titanium implant surface. The figure elucidates the various pathways by which silver disrupts/inhibits the bacterial attachment.

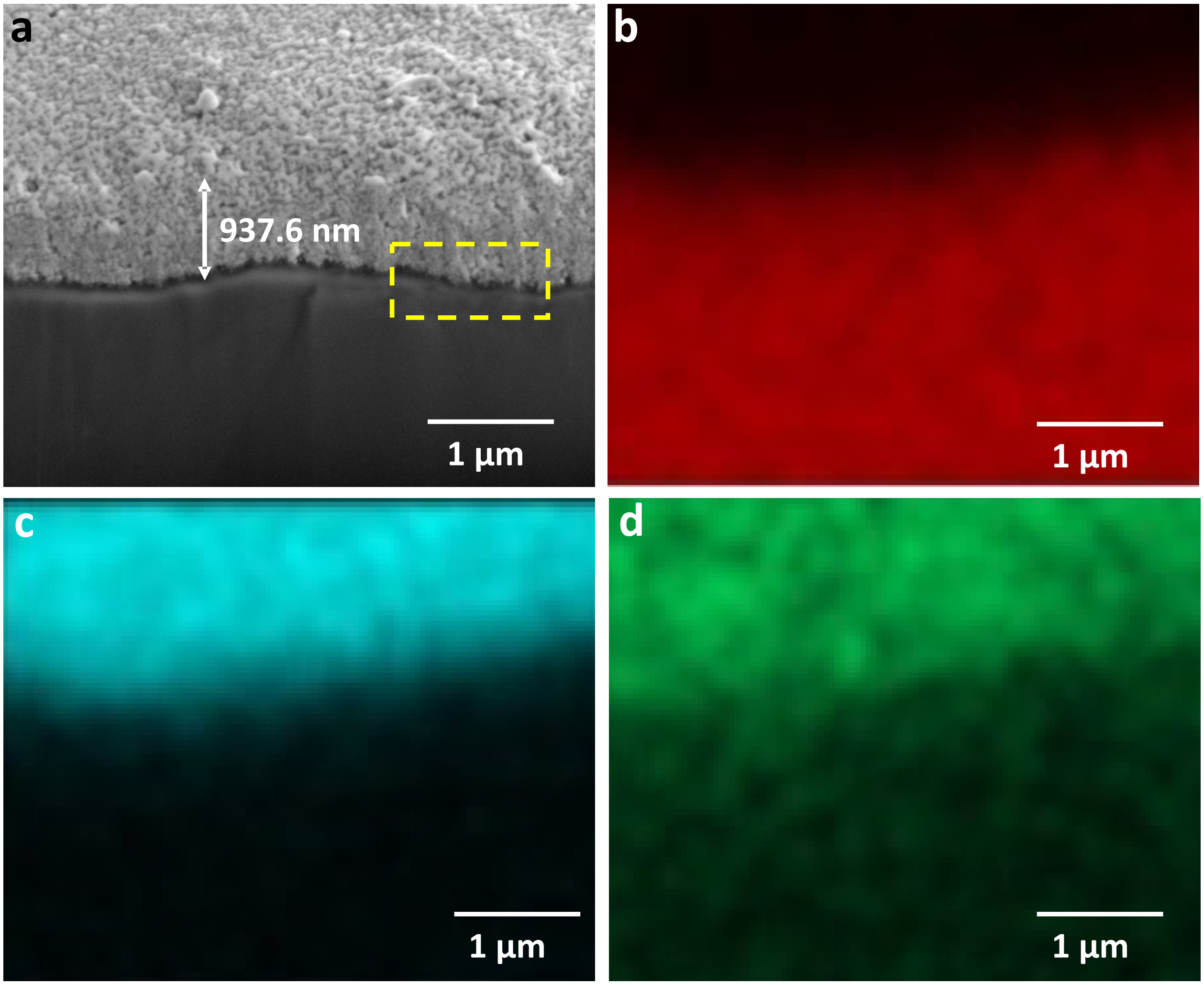

Figure S2. Surface characterization of the AgNPs spray coated onto titanium surface. (a) Cross-sectional view of the AgNPs spray coated surface, cross-sectional surface elemental mapping of (b) Ti, (c) Ag, and (d) C atoms.

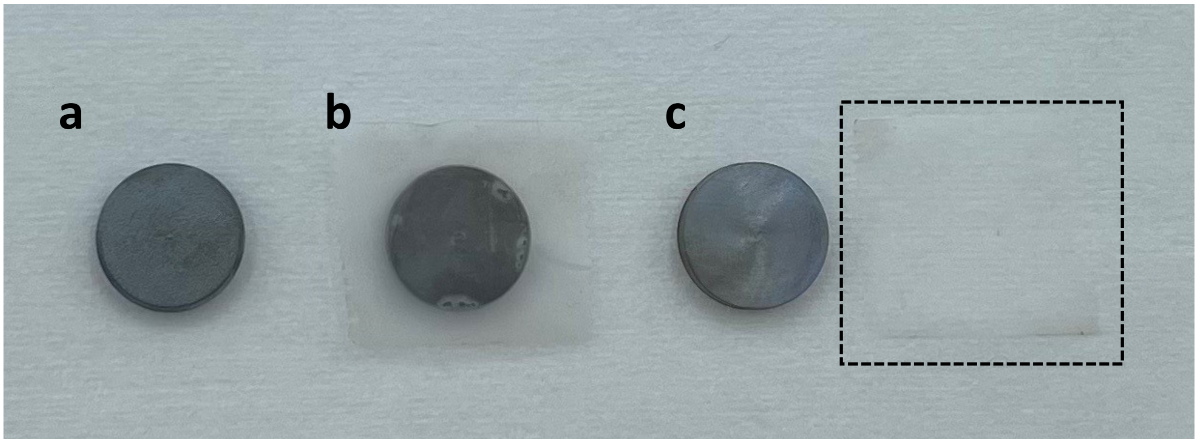

Figure S3. AgNPs adhesion test. Images showing the (a) Ag coated Ti specimen, (b) adhesive tape attachment, (c) Tape peeled off specimen with the scotch tape (dotted lines).

Table S1. Review of different strategies for laser-enabled integration of antibacterial silver composites on Ti_6Al_4V implant surfaces.

Base implant	Antibacterial Composition	Surface modification Technique	Antibacterial Efficacy	Antibacterial Stability	Bio efficacy	Long term Biosafety	Mechanical properties	Ref
**TiNi Plates**	Ag powder in PVA solution	Laser enabled surface alloying	*S. aureus* and *E. coli*	3 days	N/A	N/A	Increased surface hardness (109 %)	1
**Selective laser melted Ti_6Al_4V**	AgNPs and ZnNPs	Laser 3D printing followed by Plasma electrolytic oxidation	*MRSA*	1 day	Biocompatible with pre-osteoblasts	11 days	-	2
**Ti_6Al_4V sheets**	TiN and Ag metal targets	Antibacterial surface sputtering followed by laser texturing	*S. aureus*	1 day	Improved fibroblast cell adhesion	1 day	Increased surface roughness	3
**3D printed porous Ti_6Al_4V**	$AgNO_3$	Laser textured surface followed by dip-coating	*S. aureus* and *E. coli*	1 day	Improved pre-osteoblasts cell adhesion	5 days	Increased surface roughness	4
**Commercial Ti_6Al_4V**	Ag powder	Hot-pressing of powder followed by laser surface texturing	*P. gingivalis and P. intermedia*	3 days	N/A	-	Increased surface roughness	5
**Ti-20Zr-10Nb-4Ta alloy**	Ag foil	Physical foil pressing followed by Laser texturing	*S. aureus* and *E. coli*	1 day	Biocompatible with pre-osteoblasts	5 days	-	6
**Commercial Ti_6Al_4V**	Ag target	Silver Sputtering followed by laser surface texturing	*S. aureus*	1 day	Biocompatible with pre-osteoblasts	1 day	Microstructures surface	7
**N/A**	Ag and Ti powders	Spraying followed by *in-situ* Laser bed fusion based alloying	*S. aureus*	1 day	N/A	-	Increased tensile strength	8
**Commercial Ti_6Al_4V**	**Ag ink**	**Ag spray coating followed by laser surface alloying**	***S. aureus* and *E. coli***	**12 days**	**Bone mineralization with osteoblasts**	**21 days**	**Alloying and no increased hardness**	**Our work**

Reference:

1 Q. Qiao, V. A. M. Cristino, L. M. Tam, W. W. Chang, H. C. Qian, D. W. Zhang and C. T. Kwok, *Surf Coat Technol*, 2023, **474**, 130119.

2 I. A. J. van Hengel, N. E. Putra, M. W. A. M. Tierolf, M. Minneboo, A. C. Fluit, L. E. Fratila-Apachitei, I. Apachitei and A. A. Zadpoor, *Acta Biomater*, 2020, **107**, 325–337.

3 C. P. Priyanka, K. Keerthi Krishnan, U. Sudeep and K. K. Ramachandran, *Surf Coat Technol*, 2023, **474**, 130058.

4 Y. Jiao, X. Li, X. Zhang, G. Li, J. Fang, S. Xuan, L. Liu, S. Wang and H. Xie, *Appl Surf Sci*, 2023, **610**, 155519.

5 I. M. R. Gonçalves, E. R. Herrero, O. Carvalho, B. Henriques, F. S. Silva, W. Teughels and J. C. M. Souza, *J Biomed Mater Res B Appl Biomater*, 2021, **109**, 1588–1600.

6 X. Xue, L. Lu, D. He, Y. Guan and Y. Li, *Surf Coat Technol*, 2021, **425**, 127716.

7 F. Zou, S. Cao, Y. Luo, Z. Liu, X. Zhao, J. Hu, R. Liu, L. Cao, B. Liang, Z. Wang and Z. Weng, *Applied Physics A*, 2023, **129**, 797.

8 C. Song, Y. Chen, L. Liu, H. Lei, X. Yang, J. Hu, Q. Li, Y. Yang and Y. Li, *Mater Today Adv*, 2023, **20**, 100445.
